# Seed Carotenoid and Tocochromanol Composition of Wild Fabaceae Species Is Shaped by Phylogeny and Ecological Factors

**DOI:** 10.3389/fpls.2017.01428

**Published:** 2017-08-24

**Authors:** Beatriz Fernández-Marín, Fátima Míguez, Leire Méndez-Fernández, Agustí Agut, José M. Becerril, José I. García-Plazaola, Ilse Kranner, Louise Colville

**Affiliations:** ^1^Department of Plant Biology and Ecology, University of the Basque Country (UPV/EHU) Bilbao, Spain; ^2^Department of Zoology and Animal Cellular Biology, University of the Basque Country (UPV/EHU) Bilbao, Spain; ^3^Jardín Botánico de Olarizu Vitoria-Gasteiz, Spain; ^4^Department of Botany and Center for Molecular Biosciences, University of Innsbruck Innsbruck, Austria; ^5^Comparative Plant and Fungal Biology, Royal Botanic Gardens, Kew Ardingly, United Kingdom

**Keywords:** carotenoid, chlorophyll, cotyledon, Fabaceae, legume, Papilionoideae, tocopherol, tocotrienol

## Abstract

Carotenoids distribution and function in seeds have been very scarcely studied, notwithstanding their pivotal roles in plants that include photosynthesis and phytohormone synthesis, pigmentation, membrane stabilization and antioxidant activity. Their relationship with tocochromanols, whose critical role in maintaining seed viability has already been evidenced, and with chlorophylls, whose retention in mature seed is thought to have negative effects on storability, remain also unexplored. Here, we aimed at elucidating seed carotenoids relationship with tocochromanols and chlorophylls with regard to phylogenetic and ecological traits and at understanding their changes during germination. The composition and distribution of carotenoids were investigated in seeds of a wide range of wild species across the Fabaceae (the second-most economically important family after the Poaceae). Photosynthetic pigments and tocochromanols were analyzed by HPLC in mature dry seeds of 50 species representative of 5 subfamilies within the Fabaceae (including taxa that represent all continents, biomes and life forms within the family) and at key timepoints during seedling establishment in three species representative of distinct clades. Total-carotenoids content positively correlated with tocopherols in the basal subfamilies Detarioideae, Cercidoideae, and Dialioideae, and with chlorophylls in the Papilionoideae. Papilionoideae lacked tocotrienols and had the highest total-carotenoids, chlorophyll and γ-tocopherol contents. Interestingly, lutein epoxide was present in 72% of the species including several herbs from different subfamilies. Overall, species original from temperate biomes presented higher carotenoids and lower tocochromanols levels than those from tropical biomes. Also shrub species showed higher carotenoids content than herbs and trees. During germination, total content of photosynthetic pigments increased in parallel to changes in relative abundance of carotenoids: zeaxanthin and anteraxanthin decreased and β-carotene augmented. Notably, the highest contents of nutritionally valuable carotenoids were found in Papilionoideae subfamily to which all pulses of socio-economic importance belong. The major differences in carotenoids and tocochromanols composition across the Fabaceae are apparently related to phylogeny in conjunction with ecological traits such as biome and growth form.

## Introduction

Carotenoids are lipophilic pigments synthesized and accumulating in almost all types of plastids in most tissues and organs of photosynthetic organisms. Among the approximately 600 carotenoids described so far, six main carotenoids are commonly found in photosynthetic tissues of all species of Viridiplantae (green algae and embryophytes): lutein (L), neoxanthin (N), β-C, violaxanthin (V), anteraxanthin (A), and zeaxanthin (Z). In addition, non-ubiquitous carotenoids with more restricted distribution can also be found in chloroplasts, for example α-C, that partly replaces β-C in some species under low light (Young and Britton, 1989), and Lx, a taxonomically restricted carotenoid also related to shade-adaptation (Esteban et al., 2009b). Carotenoids play essential roles in plants as (i) accessory photosynthetic pigments, broadening the spectrum of light harvested due to their absorbance of blue and blue-green light and due to their capacity to transfer absorbed energy to Chl, (ii) in the assembly of photosystems, thereby altering structure and function of the photosynthetic apparatus, (iii) in photoprotection due to their ability to quench singlet oxygen and triplet Chl under excess light conditions and (iv) in membrane stabilization (Havaux, 1998; Dall’Osto et al., 2006, 2007; Polívka and Frank, 2010). Additionally, carotenoids play roles in pigmentation, conferring yellow to red colouration to flowers, fruits and seeds, attracting pollinators and seed dispersers (Cazzonelli, 2011). Carotenoids are also involved in phytohormone production (Demmig-Adams et al., 1996; Howitt and Pogson, 2006; Tanaka et al., 2008) as substrates for the syntheses of abscisic acid (Nambara and Marion-Poll, 2005), and strigolactones (Cazzonelli, 2011). Additionally, several apocarotenoids (molecules derived from carotenoid oxidation) such as β-ionone, or β-cyclocitral, among others, are involved in plant signaling (Auldridge et al., 2006; Ramel et al., 2013). Thus, carotenoids may play several roles of paramount importance during the initial phases of seed imbibition, germination and seedling development.

It is known that antioxidant systems play a key role in the maintenance of seed viability (Bailly and Bailly, 2004). Ascorbate and GSH are the major intracellular water-soluble antioxidants. While ascorbate is absent in mature orthodox (desiccation tolerant) seeds (Gara De et al., 1997; Tommasi et al., 1999; De Tullio and Arrigoni, 2003), GSH is maintained (Kranner and Birtić, 2005; Kranner et al., 2006) and its availability and redox state are reliable markers of seed viability (Kranner et al., 2006; Seal et al., 2010; Nagel et al., 2015). Homoglutathione, a homolog of GSH, shows a phylogenetically based distribution in Fabaceae seeds (Colville et al., 2015). The major lipophilic antioxidants are tocochromanols (tocopherols and tocotrienols) and carotenoids (Noctor and Foyer, 1998; Munné-Bosch and Alegre, 2002) and, at least in photosynthetic tissues, both are though to play synergistic protective roles against oxidative damage by preserving cell membranes (Havaux and García-plazaola, 2014), whose functionality and integrity are essential for seed viability. While tocopherol content of seeds and its critical role in maintaining seed viability has been reported (Mène-Saffrané et al., 2010; Seal et al., 2010; Sattler et al., 2013) the distribution of the main eight isoforms of tocochromanol (α-, β-, γ-, δ- tocopherols and tocotrienols) and their interaction with carotenoids in seeds still remains almost unexplored (Schroeder et al., 2006). Additionally, the scattered distribution of tocotrienols within the plant kingdom is not yet completely understood (Falk and Munné-Bosch, 2010). Together with tocochromanols and carotenoids, Chl can also sometimes be present in seeds, mostly as a remnant of incomplete degradation during seed maturation but also as part of a fully operative photosynthetic apparatus in a couple of species in which mature seeds are photosynthetic (Maroder et al., 2003). From an anthropocentric perspective, the presence of Chl in seeds is usually considered a negative aspect due to reduced seed storability [as is the case of photosynthetic seeds from *Salix* (Roqueiro et al., 2010)] and decreased marketability of seed crops [e.g., for soybean seeds with incomplete degradation of Chls (Teixeira et al., 2016)]. Nevertheless, proper assessment of its presence in relation to other plastidial molecules such as carotenoids and tocochromanols may help to broaden our understanding about the physiological meaning of Chl retention in mature seeds.

Most work on carotenoids so far dealt with photosynthetic tissues and fruits, and information available regarding their roles in seeds is scarce (Howitt and Pogson, 2006; Smolikova and Medvedev, 2015, 2016; Federico and Schmidt, 2016), with a focus on model plants such as Arabidopsis (Lindgren, 2003; Stålberg et al., 2003) and tobacco (Frey et al., 2006), and domesticated plants including oilseed crops (Bulda et al., 2008; Herchi et al., 2011; Smolikova et al., 2011); pulses (Fernández-Marín et al., 2014; Zhang et al., 2014; Ashokkumar et al., 2015) and cereals (Vallabhaneni and Wurtzel, 2009; Mamatha et al., 2011). Emphasis was laid on the nutritional value of seed crops, because carotenoids are essential to human and animal diet. Carotenes are vitamin A precursors and some xanthophylls such as L and Z are essential to prevent age-related macular degeneration (Howitt and Pogson, 2006). Furthermore, carotenoids may have anti-cancer activity (Moradzadeh et al., 2017) and some are used as food colorants, and added to cosmetics or pharmaceuticals. One of the few studies on the seeds of wild plant species revealed that grain legumes have lower contents of carotenoids (including those of nutritional value for human and livestock feeding, such as L and Z) than their wild counterparts, indicating a decrease in the concentration of carotenoids in seeds in the course of domestication (Fernández-Marín et al., 2014). These data are further supported by recent studies on seed carotenoids in the economically most important pulses: soybean, pea, chickpea, and lentil, of which the seeds of most crop genotypes contained lower total carotenoid levels than those of their wild relatives (Zhang et al., 2014; Dhakal et al., 2014; Ashokkumar et al., 2015). As suggested in Fernández-Marín et al. (2014) the loss of carotenoids during domestication likely occurred as a side-effect of the selection for other desired traits such as the loss of seed dormancy and dispersal mechanisms, and selection for seed storability and taste.

After Asteraceae and Orchidaceae, Fabaceae is the third largest angiosperm family with 765 genera and over 19000 species worldwide (Lewis, 2005; Bruneau et al., 2013; LPWG, 2017). The family includes many species of agricultural and economic importance and is well represented in temperate and tropical regions of the world, ranging in growth form from annual herbs to large trees (Rundel, 1989). Additionally, this plant family has several significant ecological roles such as (i) atmospheric nitrogen fixation through symbiotic associations with bacteria and/or fungi (Clarke and Gorley, 2006), and (ii) provision of highly nutritious food for animals. In recognition of the importance of legumes for agriculture and human and livestock nutrition (Foyer et al., 2016), the United Nations declared the year 2016 as the International Year of Pulses. The Fabaceae family has traditionally been classified into three subfamilies: Caesalpinioideae DC. and Mimosoideae DC. (mostly represented by trees and distributed mainly in the tropics), and Papilionoideae DC. (mostly represented by herbs and shrubs and distributed mainly in temperate regions), with Caesalpinioideae long-recognized as a non-monophyletic group (Lewis, 2005; Bruneau et al., 2013). Just very recently the Legume Phylogeny Working Group (LPWG) has reclassified the Fabaceae into six subfamilies, which include (i) the segregation of former Caesalpinioideae into four new subfamilies (Detarioideae, Cercidoideae, Duparquetioideae, and Dialioideae), (ii) the unification of Caesalpinioideae *sensu stricto* with the former subfamily Mimosoideae, now included as a clade pending classification within Caesalpinioideae and (iii) the unchanged Papilionoideae subfamily (LPWG, 2017).

Here, the composition and distribution of carotenoids were investigated in seeds of a wide range of wild Fabaceae species, representing 5 of the 6 subfamilies according to current classification from the LPWG (2017) and including taxa that represent all continents, biomes and life forms within Fabaceae (only the monospecific subfamily Duparquetioideae was not included in this study). We aimed (1) to provide an comprehensive overview of carotenoid composition and distribution in wild legume seeds, (2) to determine whether seed carotenoid composition and content relate to tocochromanol composition and content and/or to Chl retained after seed maturation, have a phylogenetic basis, and/or have an ecological basis. Additionally, (3) changes in photosynthetic pigments (content and composition of Chls and carotenoids) were assessed upon germination of three selected species (each from distinct clade).

## Materials and Methods

### Plant Material

Seeds of 50 wild species were obtained from the Royal Botanic Gardens, Kew, United Kingdom, and from the Banco de Germoplasma del Jardín Botánico de Olarizu de Vitoria (JBO) (Spain). Seed material was silica gel-dried and stored at -80°C before the analyses. The basal subfamilies, Cercidoideae, Detarioideae, and Dialioideae, which are smaller in terms of species number, were grouped for some of the analyses and are referred to as “other Fabaceae,” Details for the 50 species are provided in Supplementary Table S1. Prior to analyses, dry seeds were ground to a fine powder with mortar and pestle under liquid nitrogen. Whenever possible, five replicates of at least *n* ≥ 5 seeds each were prepared from each species (exceptions with insufficient material available are specified in Supplementary Table S1).

### Carotenoid and Chlorophyll Analyses

Around 20 mg DW of seeds (exact weight recorded) was used for the analyses of photosynthetic pigments. Carotenoids and chlorophylls were extracted from seeds powder at ≤4°C using 0.25 mL of 95% (v/v) aqueous acetone solution, followed by centrifugation for 10 min at 16100 *g*. The supernatant was preserved, the pellet resuspended in 0.25 mL of pure acetone and centrifuged for 10 min at 16100 *g*. The two supernatants were mixed and filtered through a 0.2 μm PTFE filter (Teknokroma, Barcelona, Spain). Photosynthetic pigments were separated by HPLC on a reversed-phase C18 column (Waters Spherisorb ODS1, 4.6 mm × 250 mm, Milford, MA, United States) and detected with a photodiode array detector, following the method of García-Plazaola and Becerril (1999, 2001). As described in García-Plazaola and Becerril (1999, 2001), carotenoids absorbance was analyzed at 445 nm, and their identification and quantification performed with commercial standards.

### Tocochromanol Analyses

Around 20 mg of DW of seeds (exact weight recorded) was used for tocochromanol analyses. Tocochromanols were extracted at ≤4°C from seed powder using 0.5 mL of pure heptane, followed by centrifugation for 10 min at 16100 *g*. The supernatant was preserved, the pellet resuspended in 0.5 mL of pure heptane and centrifuged for 10 min at 16100 *g*. The two supernatants were mixed and filtered through a 0.2 μm PTFE filter (Teknokroma, Barcelona, Spain). Tocochromanols were separated by HPLC on a diol column (Supelcosil LC-Diol, 4.6 mm × 250 mm, 5 μm particle size, Supelco Analytical, Sigma–Aldrich, Bellefonte, PA, United States). The system was operated with an eluent of heptane:tert-butylmethyl ether (97.5:2.5, v:v) at a flow rate of 1 mL per min and tocochromanols were detected by a fluorescence detector Waters 474 (Waters, Barcelona, Spain) with an excitation wavelength of 295 nm and emission wavelength of 330 nm. Calibration was performed using commercial standards of α-, β-, γ-, and δ- tocopherols and tocotrienols (Calbiochem, Darmstadt, Germany).

### Phylogeny, Plant Traits and Ecological Parameters

A cladogram showing the phylogenetic relationships of the 50 species used in this study was generated from Bayesian phylogenetic consensus tree data published by LPWG (2017) and available from Dryad Digital Repository ^1^, which was pruned and annotated using the Interactive Tree Of Life (iTOL) v3 (Letunic and Bork, 2016).

Information on Raunkiaer’s life form, leaf phenology, and natural habitat and geographical distribution of the species (when available) were obtained from ILDIS (International Legumes Database and Information Service^2^) and otherwise from Useful Tropical Plants Database^3^ 2017, World Agroforestry Center^4^, Flora Ibérica^5^ and/or (Aizpuru et al., 1999). Based on this geographical information, climates were assigned to each species according to the updated World Map of Köppen–Geiger Climate Classification (Kottek et al., 2006). All diverse habitats were grouped into four main floristic biomes. All these compiled ecological traits and the categories (groups) therein are shown in Supplementary Table S1.

### Germination Experiment

For analysis of carotenoids during seed germination, three species from three different clades were chosen: *Erythrophleum africanum* (Caesalpinioideae), *Leucaena leucocephala* (Mimosoideae in the previous classification, and currently Mimosoid clade) and *Podalyria myrtillifolia* (Papilionoideae) (Supplementary Table S1). Five biological replicates of 4–10 seeds were used for each species. Seeds were chipped to remove physical dormancy and germinated on Petri dishes with three layers of wet filter paper. Conditions during imbibition and germination were 23/15°C and 40 μmol photons m^-2^ s^-1^ of Photosynthetic Photon Flux Density (PPFD) during a 14/10 h day/night photoperiod. Five time-points were established for sampling during germination and early stages of seedling development as follows: *Stage* (1): dry seeds, *Stage* (2): 48 h of germination, *Stage* (3): radicle had emerged, radicle length < seed length, *Stage* (4): radicle length > seed length, *Stage* (5): hypocotyl length > seed length. Photosynthetic pigments were analyzed in the cotyledons.

### Statistics

One-way ANOVA followed by Duncan’s *post hoc* test was used to test for differences in the content of metabolites among subfamilies and among categories within each ecological factor after checking homoscedasticity of data. Some parameters showed heteroscedasticity even after data transformation. In these cases, the non-parametric Kruskal–Wallis *H*-test was applied, followed by multiple pairwise comparison with Kolmogorov–Smirnov *Z*-test. Pearson’s correlation (or Spearman for non-parametric data) was applied to analyze the relationship between carotenoid and tocopherol contents, between Chl and carotenoid contents, and between Chl and tocochromanol contents. When correlation was significant, a linear regression model was applied. These statistical analyses were conducted using the SPSS 24.0 statistical package at a significance level of α = 0.05.

In addition, multivariate analyses were conducted using PRIMER 6 (Clarke and Gorley, 2006) software. Data were transformed with Log (X+1) and standardized, and euclidean distance was used to build up resemblance matrices. A Principal Coordinate Analysis (PCOA) examined the dominant patterns of intercorrelation among metabolite concentrations in different species. Twelve variables were included in the analysis, some of them being the sum of individual metabolites: N, V, Lx, A, L, Z, β-C, α-T, γ-T, (β+δ)-T, TotChl and TotT3. Several factors were tested to evaluate the relevance of phylogeny or ecological traits to metabolite composition and content. Both, the former (Lewis, 2005) and the new (LPWG, 2017) subfamily classifications of the Fabaceae were tested as phylogenetic factors. Different ecological traits were used as ecological factors (details in Supplementary Table S1) and site grouping underlying these factors was evaluated by ANOSIM [Analysis of Similarity: (Clarke, 1993)]. This routine is based on a non-parametric permutation procedure applied to the similarity matrix underlying the classification of samples, and is an absolute measure of differences between groups in the multidimensional space. The ANOSIM R statistic varies from zero to one, indicating a degree of discrimination between groups (up to maximum degree of discrimination when *R* = 1). The significance level was considered α = 0.05 after 9999 permutations. Finally, the contribution of the metabolites to the between-groups dissimilarities was assessed by SIMPER (Similarity Percentages).

## Results

### Carotenoid, Tocochromanol and Chlorophyll Composition in Fabaceae Seeds

**FIGURE 1:**
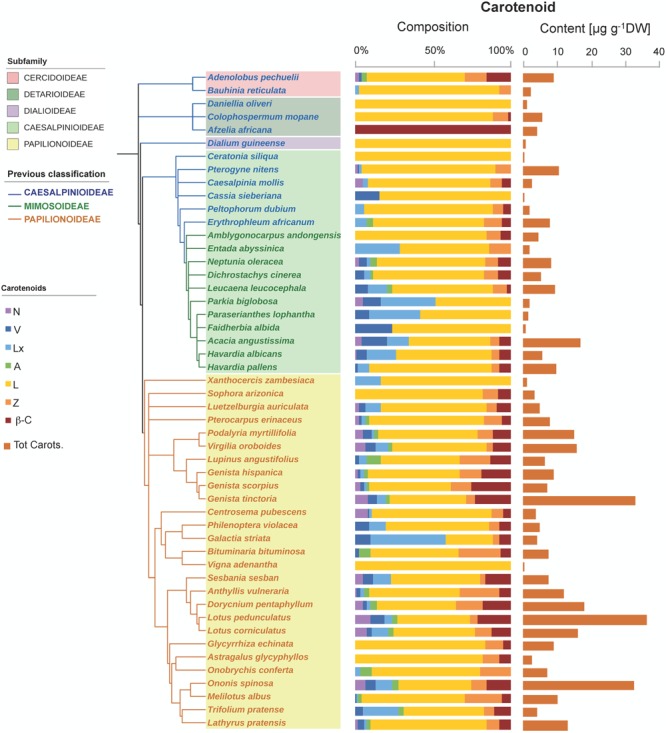
Carotenoid composition and total carotenoid contents in seeds of 50 species across the Fabaceae family. Subfamilies are depicted in shaded colors according to the new classification of Fabaceae (LPWG, 2017). Former subfamilies (previous classification) according to Lewis (2005) are additionally indicated in the cladogram by the color of lines and species’ names. Each value represents the mean of *n* ≥ 5 replicates; exceptions are given in Supplementary Table S1.

The six major carotenoids usually found in photosynthetic tissues were also detected in Fabaceae seeds, but only 26% of the species analyzed contained the whole set, with most species having 2–4 carotenoids only (**Figure 1**). Lutein, Z, and β-C were the most frequent carotenoids, occurring in 98, 80, and 74% of the species analyzed, respectively; and were also found at the highest contents; on average this was 4.8 ± 0.6, 0.8 ± 0.1, and 1.0 ± 0.2 μg carotenoid g^-1^ DW for L, Z, and β-C, respectively (**Figure 1** and Supplementary Figure S1). Lutein was absent only in one of the analyzed species: *Afzelia africana*, which contained β-C as the only carotenoid (**Figure 1**). Neoxanthin and A were the least frequent carotenoids present in less than 50% of the species (Supplementary Figure S1A). The average total carotenoids content of the 50 Fabaceae species was 8.1 ± 1.1 μg g^-1^ DW (Supplementary Figure S1B). Three species (*Genista tinctoria, Lotus corniculatus*, and *Ononis spinosa*) showed remarkably high contents of total carotenoids: 33.1 ± 4.0, 36.4 ± 1.3, and 32.7 ± 0.7 μg g^-1^ DW, respectively, whereas the rest of the analyzed species contained less than 20 μg g^-1^ DW (**Figure 1**). Surprisingly, lutein epoxide (Lx), which is only present in the photosynthetic tissues of a few species with scattered phylogenetic distribution (Esteban et al., 2009a) was remarkably frequent: it was found in 72% of the analyzed species (**Figure 1** and Supplementary Figure S1).

**FIGURE 2:**
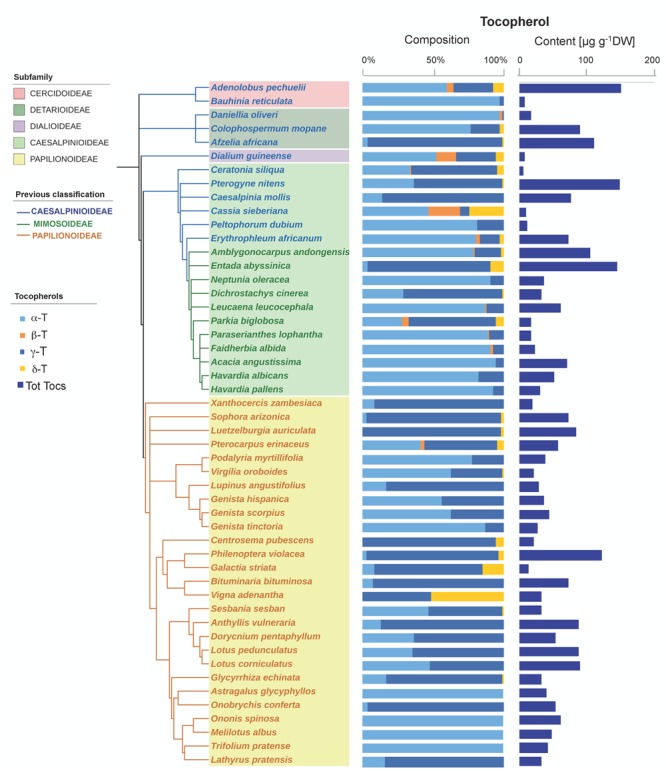
Tocopherol composition and contents in seeds of 50 species across the Fabaceae family. Subfamilies are depicted in shaded colors according to the new classification of Fabaceae (LPWG, 2017). Former subfamilies (previous classification) according to Lewis (2005) are additionally indicated in the cladogram by the color of lines and species’ names. Each value represents the mean of *n* ≥ 5 replicates; exceptions are given in Supplementary Table S1.

**FIGURE 3:**
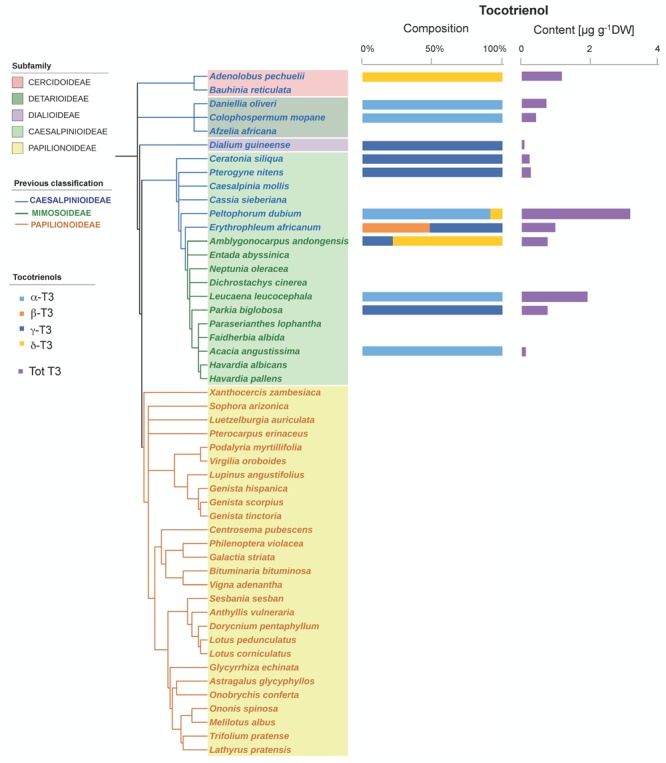
Tocotrienol composition and contents in the seeds of 50 species across the Fabaceae family. Subfamilies are depicted in shaded colors according to the new classification of Fabaceae (LPWG, 2017). Former subfamilies (previous classification) according to Lewis (2005) are additionally indicated in the cladogram by the color of lines and species’ names. Each value represents the mean of *n* ≥ 5 replicates; exceptions are given in Supplementary Table S1.

Tocochromanols were present at higher concentrations than carotenoids (one order of magnitude on average, **Table 1**). The main eight tocochromanols α-, β-, γ-, δ-tocopherols (T) and α-, β-, γ-, δ-tocotrienols (T3) were also found in the Fabaceae seeds analyzed. All species contained tocopherols, with α-T and γ-T being the most common isoforms, present in 96 and 92% of the species, respectively (**Figure 2** and Supplementary Figure S2). These two isoforms were also the most abundant across the 50 species, with mean concentrations of 23.6 ± 3.3 (SE) and 28.0 ± 4.7 μg g^-1^ DW, respectively. On the other hand, tocotrienols were found in 24% of the species only (**Figure 3** and Supplementary Figure S2), at much lower concentrations (ranging from 0 to 3.1 μg g^-1^ DW) than those of total tocopherols (7.1 to 151.3 μg g^-1^ DW) (**Figures 2, 3**). Carotenoid and tocopherol contents were positively correlated in the Fabaceae (Spearman correlation coefficient = +0.435, *P* = 0.002), but the correlation was significant only in the basal clade “Other Fabaceae” (Pearson correlation coefficient = +0.932, *P* = 0.01) (**Table 2** and Supplementary Figure S3).

**Table 1:** Average seed content of total carotenoids (Tot Carots.), total tocochromanols (Tot Tocochrom.), tocopherols (Tot T), tocotrienols (Tot T3), and total chlorophylls (Tot Chl) in main clades of the Fabaceae.

Subfamily		Tot				
*(or clade)*	Tot Carots.	Tocochrom.	Tot T	Tot T3	Tot Chl	Chl a/b
Other Fabaceae	3.8 ± 1.3	64.2 ± 25.1	63.8 ± 25.0	0.4 ± 0.2a	4.7 ± 1.5ab	1.4 ± 0.2
Caesalpinioideae	3.6 ± 0.9	54.6 ± 13.2	54.1 ± 13.1	0.5 ± 0.1a	3.6 ± 0.9a	2.0 ± 0.5
*Mimosoid*	*5.9 ± 1.5*	*54.2 ± 12.1*	*53.9 ± 12.0*	*0.3 ± 0.2*	*4.4 ± 0.8*	*2.1 ± 0.2*
Papilionoideae	10.8 ± 1.8	50.3 ± 5.2	50.3 ± 5.2	ndb	22.2 ± 5.5b	2.0 ± 0.1

*Minoritary subfamilies Cercidoideae, Detarioideae and Dialioideae are grouped as “Other Fabaceae.” Data for the clade Mimosoid are also shown: no significant differences between Mimosoid and non-Mimosoid species were found within Caesalpinioideae. Units are μg of metabolite per g DW of seed. Values are means ± SE of all the species analyzed per clade: Other Fabaceae (*n* = 6 species), Caesalpinioideae (*n* = 17), *Mimosoid* (*n* = 11), *Papilionoideae* (*n* = 27). Significant differences among subfamilies are indicated with letters (*P* < 0.05).*

**Table 2:** Pearson (or Spearman) correlation coefficients between lipophilic antioxidant and pigment contents of Fabaceae seeds.

Correlation	Fabaceae	Other Fabaceae	Caesalpinioideae	*Mimosoid*	Papilionoideae
Tot Carots. Vs. Tot T	**(+0.435)^∗∗^**	**+0.932^∗∗^**	+0.362	*+0.05*	+0.427
Tot Chl vs. Tot Carots.	**(+0.624)^∗∗∗^**	+0.220	+0.441	*+0.056*	**+0.780^∗∗∗^**
Tot Chl vs. Tot T	-0.320	+0.095	-0.162	-***0.679^∗^***	+0.226

*Significant correlations are indicated in bold font. Asterisks depict the level of significance: ^∗^*P* < 0.05, ^∗∗^*P* < 0.01, *P* < 0.001.*

**FIGURE 4:**
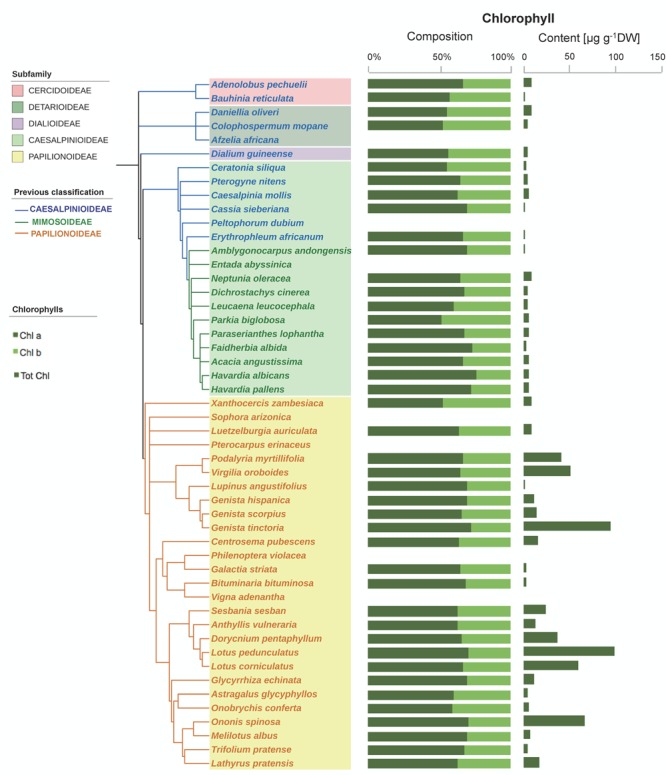
Chlorophyll composition and contents in the seeds of 50 species across the Fabaceae family. Subfamilies are depicted in shaded colors according to new classification of Fabaceae (LPWG, 2017). Former subfamilies (previous classification) according to Lewis (2005) are additionally indicated in the cladogram by the color of lines and species’ names. Each value represents the mean of *n* ≥ 5 replicates; exceptions are given in Supplementary Table S1.

**FIGURE 5:**
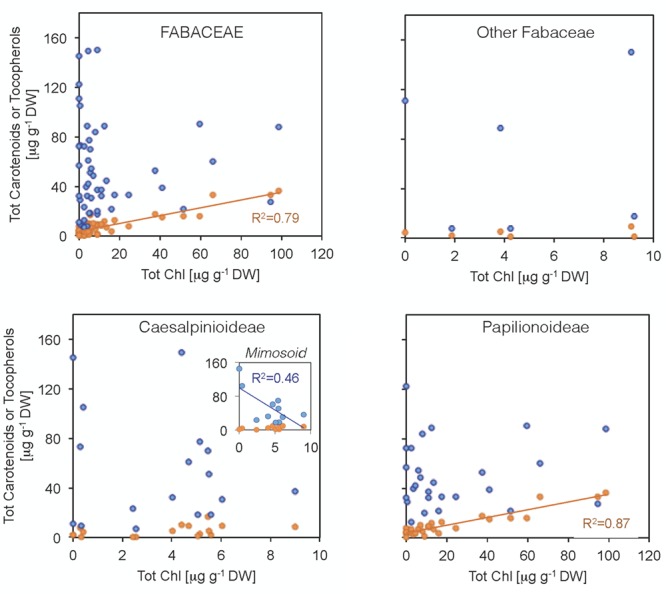
Relationship between total chlorophyll contents and (i) carotenoid (orange) or (ii) tocopherol (blue) contents in Fabaceae seeds. Each data point represents the mean value for a single species. Carotenoid and chlorophyll contents were positively correlated within the Fabaceae family due to strong correlation in the Papilionoideae subfamily. Tocopherol and chlorophyll contents were negatively correlated for the Mimosoid clade within the Caesalpinioideae. Fits a linear regression model and *R*^2^ values are shown for these cases (*P* < 0.05).

Some Chl was found in a high proportion of the seeds (76% of the species, showing an average Chl a/b ratio of 1.9, **Figure 4**). Although Chl was mostly found at low concentrations (14 μg g^-1^ DW on average), a few species had higher contents, particularly among Papilionoideae (i.e., 10 species in this subfamily showed values >14 μg g^-1^ DW; **Figure 4**). Total carotenoid content was positively correlated with total Chl content in Fabaceae (**Figure 5**). When plotted separately by subfamily, it was found that this correlation was a consequence of a strong correlation in the subfamily Papilionoideae (data fitted a linear regression model, *R*^2^ = 0.87, *P* < 0.001), which showed the highest carotenoid and Chl contents (**Table 2** and **Figure 5**). In the Mimosoid clade, total tocopherols correlated negatively with total Chl content (data weakly but significantly fitted a linear regression model, *R*^2^ = 0.46, *P* = 0.022) (**Figure 5**).

### Phylogenetic Patterns of Photosynthetic Pigments and Tocochromanols

**FIGURE 6:**
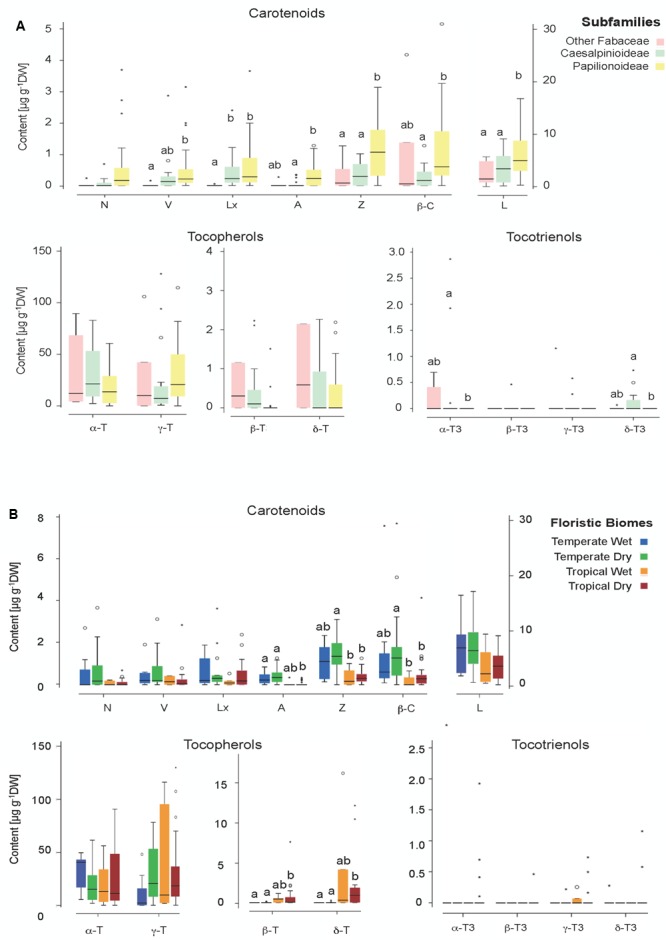
Box plot illustrating the contents of individual metabolites for **(A)** different phylogenetic groups (subfamilies) and **(B)** different floristic biomes (details in Supplementary Table S1). Boxes cover 50% of the data. Central lines represent the medians and whiskers represent the minimum and maximum values among non-atypical data. When significant, differences among categories are indicated with letters (*P* < 0.05).

**FIGURE 7:**
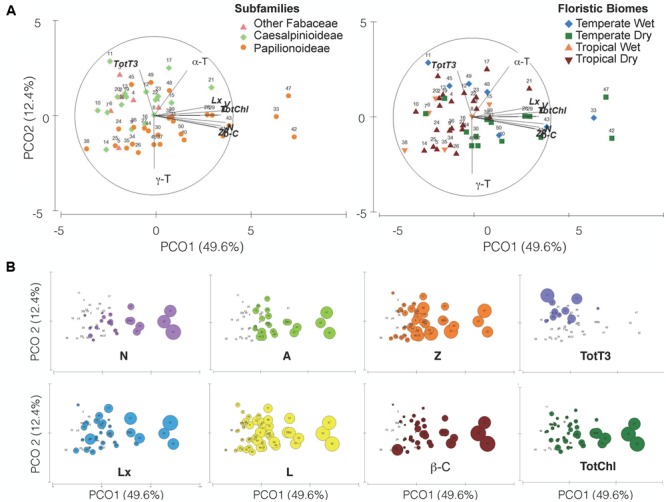
Principal Coordinate Analysis ordination plot of the 12 variables analyzed in the 50 species studied (detailed in **Table 1** and Supplementary Table S1). Variables that showed Pearson’s correlations coefficient (*r* > 0.5) with the PCOA axes are shown as overlay vectors. **(A)** Colored symbols identify subfamilies (left) or floristic biomes (right). **(B)** Same PCOA ordination but with superimposed bubbles representing the concentration of the eight variables (metabolites) that most contributed to the spatial ordination of the species. The size of the bubbles is proportional to the concentration of the metabolite within each panel.

A phylogenetic pattern regarding the contents and distribution of photosynthetic pigments and tocochromanols in Fabaceae seeds was revealed by both univariate and multivariate analyses (**Figures 6, 7, Tables 1, 2**, and Supplementary Table S3). Papilionoideae was the most distinct subfamily, containing the highest amounts of total carotenoids and Chl (**Table 1** and **Figures 1, 6A, 7A**). Papilionoideae was the only subfamily with γ-T as the main isoform of tocopherol (**Figures 2, 6A**) and which lacked tocotrienols (**Table 1** and **Figure 3**).

The PCOA defined two principal components (**Figure 7**) that together explained 62.1% of the accumulated variance (PCo1 = 49.7%; PCo2 = 12.4%). PCo1 defined a gradient in carotenoid and TotChl content (Pearson’s correlation with PCo1: *r* ≥ 0.80, Supplementary Table S2), where the species 33 (*G. tinctoria*), 42 (*L. corniculatus*) and 47 (*O. spinosa*) had the highest carotenoid and TotChl concentrations (**Figure 7B**), toward lower values (or the absence of some metabolites, i.e., N, V, A, **Figure 7B**) on the opposite side of the PCO1. The PCo2 component defined a gradient in tocochromanols that distinguished mainly the three subfamily levels considered (**Figure 7A**). For instance, higher tocotrienol contents were found in the species from the most basal clades “Other Fabaceae” and Caesalpinioideae (Pearson’s correlation with PCo2: α-T *r* = 0.64; γ-T *r* = -0.78; TotT3 *r* = 0.58) (Supplementary Table S2). From that, it can be observed that the PCo1 explained variation in the common metabolites found in the seeds and PCo2 can be related with a gradient in rare metabolites within subfamilies.

The differences in metabolite concentration among subfamilies in the multivariate space were weak but significant (ANOSIM Global *R* = 0.104, *p* = 0.045). The differences were mainly due to the different metabolite contents between Papilionoideae and Caesalpinioideae subfamilies (*R* = 0.104, *p* = 0.033; Supplementary Table S3). SIMPER analysis (Supplementary Table S3) revealed that the five metabolites that contributed to almost 50% dissimilarities between Caesalpinioideae and Papilionoideae subfamilies were, in decreasing order, Tot-T3 (10.96%), A (8.83%), TotChl (8.81%), Z (8.81%), and L (8.30%). The previous classification (Lewis, 2005) did not show a global significant difference (Supplementary Table S3), so it was not further analyzed by multivariate analyses. Univariate analyses already revealed similar tendencies at those found for new classification: major differences were found between Papilionoideae and the more basal clades, mainly driven by differences in the contents of V, Lx, Z, β-C and L (Supplementary Figure S4A).

### Ecological Factors That Influence Photosynthetic Pigments and Tocochromanols in Seeds

Both univariate and multivariate analyses also revealed influences of ecological factors on the contents and distribution of photosynthetic pigments and tocochromanols in Fabaceae seeds (**Figures 6B, 7A** and Supplementary Figure S4B). Among all ecological factors tested (Supplementary Table S1) three of them, functional groups, life form and floristic biomes, showed weak but significant importance in explaining metabolite contents of wild Fabaceae seeds (ANOSIM Global *R* = 0.166–0.223, *p* < 0.05; Supplementary Table S2). Pairwise comparisons within “functional groups” showed that trees differed from shrubs (*R* = 0.348) and from herbs (*R* = 0.144). Within “life forms” phanerophyte was the most distinct group, differing from chamaephyte (*R* = 0.226) and from hemicryptophyte (*R* = 0.245). In the case of “floristic biomes,” tropical ecosystems with seasonal rain (TropDry) differed from temperate systems with a dry season (TemDry) (*R* = 0.226) and from temperate nemoro-oceanic ecosystems (TemWet) (*R* = 0.345). Overall, woody and/or tropical characters appeared as the most differentiating drivers among ecological traits.

The contribution of metabolites related with the ecological traits that accounted up to almost 50% of dissimilarity percentages among categories, were somewhat different between functional group, life form and floristic biome (Supplementary Table S3). The pair TropDry and TempWet was best discriminated by TotT3, a metabolite accounted with the highest value of dissimilarity contribution (13.52%) among all tested group differences (Supplementary Table S3). TotT3 appeared as the distinguishing metabolite only in this case; β-C, N, Z, and α-T were the most relevant for the differences among functional groups, while β-C, Z, and A, contributed mostly to differences among life forms (Supplementary Table S3).

### Changes in Carotenoids during Germination and Early Seedling Growth

**FIGURE 8:**
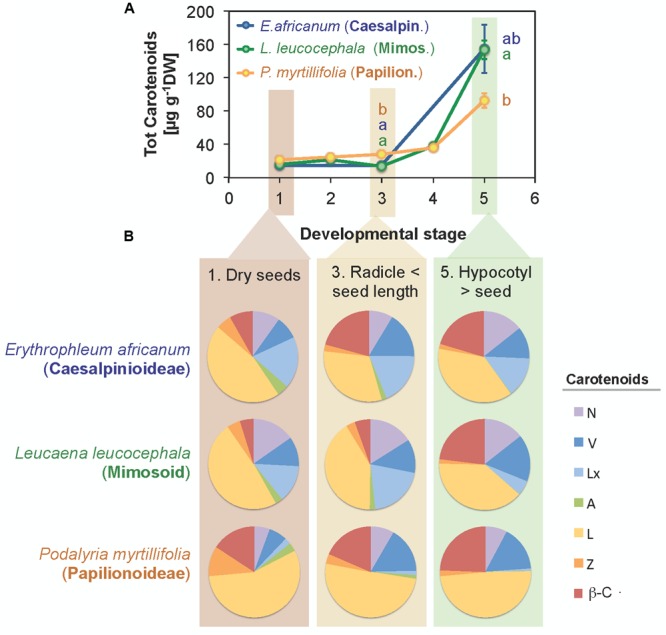
Changes in total carotenoid contents **(A)** and composition **(B)** of cotyledons during germination of seeds of three selected species. One species representative of each subfamily within the Fabaceae was analyzed. Data are mean ± SE (*n* = 5). Significant differences in carotenoid contents between species within a developmental stage are indicated with letters.

Changes in carotenoids were followed during germination and the early stages of seedling development in three species, *P. myrtillifolia* (Papilionoideae), *L. leucocephala* (Mimosoid clade, Caesalpinioideae) and *E. africanum* (Caesalpinioideae). During germination, total carotenoid contents increased slightly in *P. myrtillifolia* seeds only, in which it rose 1.4-fold from the initial level in dry seeds (*Stage* 1, according to Materials and Methods) to the end of germination (*Stage* 3) (**Figure 8A**). After germination was completed at *Stages* 4 and 5 (**Figure 8**) carotenoid contents steeply increased in all three species, reaching levels up to 10-fold higher than the initial total carotenoid contents by *Stage* 5 (hypocotyl length > seed length). This was particularly remarkable for the two Caesalpinioideae species, with 7.6-fold increase in *L. leucocephala* and a 10.7-fold increase in *E. africanum*. This occurred in parallel with an even faster increase in Chl content (Supplementary Figure S5A) as the cotyledon transformed into a photosynthetic organ. Interestingly, the increase in total carotenoids was accompanied by a parallel change in composition (**Figure 8B** and Supplementary Figure S5B): the relative abundance of β-C and V to total carotenoids increased, whereas the relative abundance of xanthophylls such as Z and A decreased in the three species (**Figure 8B** and Supplementary Figure S5B).

## Discussion

### Photosynthetic Pigments in Fabaceae Seeds: Features and Potential Roles

This study provides a comprehensive report on the distribution of photosynthetic pigments and tocochromanols in the seeds of wild species in the Fabaceae family. The carotenoid composition of Fabaceae seeds (**Figure 1**) resembled that typical of leaves (Esteban et al., 2015), although only 26% of the species contained the full set of photosynthetic carotenoids. However, the total carotenoid content (8.1 ± 1.1 SE μg g^-1^ DW on average) was lower by three orders of magnitude than that found in photosynthetic tissues (Esteban et al., 2015) and in the range found in seeds of other plant groups, e.g., 0.5–3.9 μg g^-1^ of seed DW have been reported for seed of several crops including *Brassica oleracea, B. napus, Vicia sativa* and several cereals (Bulda et al., 2008; Mamatha et al., 2011) and in cultivated species from the Fabaceae family approximately 10 μg g^-1^ DW have been reported for different species and genotypes of pulses (Fernández-Marín et al., 2014; Ashokkumar et al., 2015).

Lutein was the most abundant carotenoid in Fabaceae seeds (**Figure 1** and Supplementary Figure S1). This is a general rule in photosynthetic tissues, but also in seeds and/or cotyledons from other families such as Cucurbitaceae (Matus et al., 1993; Esteban et al., 2009a), Solanaceae (Frey et al., 2006), Poaceae and Brassicaceae (Vallabhaneni and Wurtzel, 2009; Mamatha et al., 2011; Smolikova et al., 2011). The high frequency of Lx, which was present in 72% of the species analyzed (Supplementary Figure S1) was surprising. Lutein-epoxide is an infrequent carotenoid with a scattered phylogenetic distribution, the presence of which in leaves has been related to the operation of a photoprotective cycle (LxL cycle). The de-epoxidation of Lx to L in this cycle contributes to the harmless dissipation of energy in the photosynthetic apparatus of shade leaves (Matsubara et al., 2007; Esteban et al., 2009b). Within the Fabaceae family its presence was previously reported in leaves of *Inga* sp. (Matsubara et al., 2005) and in a few wild relatives of pulses: *Glycine soja, Vigna unguiculata*, and *Lathyrus cicera* (Fernández-Marín et al., 2014). So far, significant amounts of Lx were considered typical of only several unrelated woody species, with a few exceptions, among herbaceous non-parasitic plants such as *Cucumis sativus* (Esteban et al., 2009a). Our study provides evidence for a wider distribution of Lx among several herbaceous Fabaceae species of different subfamilies: *Neptunia oleracea* (Caesalpinioideae), and nine further herbaceous species among Papilionoideae (**Figure 1** and Supplementary Table S1). As described previously for cucumber (Esteban et al., 2009a), the Lx/Chl ratio decreased in the cotyledons of the three selected species used to study germination and seedling establishment from 1.12 to 0.04 g g^-1^ Chl in *E. africanum*, from 0.23 to 0.01 g g^-1^ Chl in *L. leucocephala*, and from 0.013 to 0.003 g g^-1^ Chl in *P. myrtillifolia* (data derived from initial and final *stages* in **Figure 8A**; also note the visible decrease in the proportion of Lx to total carotenoids ratio in **Figure 8B**). As suggested for cucumber (Esteban et al., 2009a), Lx could be of relevance for the initial stages of germination in epigeous cotyledons, such as those of the three selected Fabaceae species, where it could help maximizing light harvesting efficiency, and is progressively converted to L (that can additionally play a photoprotective role) as cotyledons develop into fully photosynthetic organ toward an environment of higher irradiance.

Related to this, during seedling development of the three selected species the composition of photosynthetic pigments shifted toward the typical composition of a fully photosynthetic leaf (**Figure 8B**). As an example, the Chl a/b ratio increased, and the ratio of most carotenoids to total Chl decreased (data not shown). The decrease in the Z abundance relative to total carotenoids was particularly remarkable (**Figure 8B**). The higher relative abundance of Z in dry seeds could indicate a protective role for this carotenoid in plastid membranes of dry seeds, similar to that reported for desiccated photosynthetic tissues (Fernández-Marín et al., 2009, 2011, 2013), which becomes progressively less important during and post germination.

An antioxidant role of seed carotenoids could be particularly relevant in those species retaining higher amounts of Chl upon maturation (Smolikova et al., 2011). Accordingly, a positive correlation between Chl and carotenoid content was found in Papilionoideae (**Figure 6**), the subfamily with the greatest number of species containing relatively high amounts of Chl (22 μg g^-1^ DW on average, **Table 1**). This content is still far lower than that of photosynthetic seeds such as those of *Salix nigra*, which was reported to contain 1373 mg 100 g^-1^ DW (Roqueiro et al., 2010), which is much higher than the average Chl content of a leaf (Esteban et al., 2015). Although carotenoids are generally degradated during seed maturation (Gonzalez-Jorge et al., 2016), our data, together with the fact that carotenoids (particularly xanthophylls) are required for a functional PSII assembly (Markgraf and Oelmüller, 1991; Dall’Osto et al., 2007) may indicate that carotenoids are retained during legume seed maturation to (i) support cellular integrity in the dry state, and (ii) provide a basis for rapid development of a functional photosynthetic chloroplast upon germination. In that sense, this work points out the need for future investigation in that direction.

### Lipophilic Antioxidants in Fabaceae Seeds: Ecological and Phylogenetic Basis for Their Distribution and Contents

The Fabaceae family is particularly abundant and diverse in seasonally dry tropical forests and in temperate-xeric shrublands (Wojciechowski et al., 2004), i.e., more than 50% of the Fabaceae species grow in semiarid or seasonally dry tropical biomes (Lewis, 2005). Fabaceae species are markedly less abundant in mesic temperate habitats (i.e., less than 40% of the species grow in temperate regions (Lewis, 2005)), including the understorey of cool temperate forests and many arctic and alpine regions (Wojciechowski et al., 2004). Their higher abundance in semi-arid to arid habitats has been related to their nitrogen-demanding metabolism, which is considered an adaptation to unpredictable or variable climatic conditions where leaves must be produced opportunistically (McKey, 1994). Particularly the most basal clades (i.e., Caesalpinioideae and those termed “other Fabaceae” in this study) are well represented in tropical environments while very poorly represented in temperate biomes (i.e., less than 3% of the species from any of those subfamilies according to Lewis (2005). Related to the remarkable properties of Fabaceae species to establish symbiosis with nitrogen-fixing microorganisms, recent advances in carotenoid biochemistry provide increasing evidence for a significant role for carotenoids in plant signaling, which is of potential relevance for interactions between plants and soil microorganisms. Volatile organic compounds such as β-cyclocitral and β-ionone, which act as attracting odors for microorganisms, are produced from β-C either after its oxidation by ^1^O_2_ (Ramel et al., 2013) or by the action of cleavage dioxygenases (CCDs) (Simkin et al., 2004). In addition, strigolactones, which are best known for their roles in establishment of mycorrhizal and nitrogen-fixing symbioses [e.g., they increase *Sinorhizobium* swarming motility (Peláez-Vico et al., 2016)] are also synthesized from β-C by CCDs (Al-Babili and Bouwmeester, 2015; Bruno et al., 2016). All these facts considered, it could be reasonable to expect that carotenoid composition and/or contents in Fabaceae seeds are related to their bioclimatic region of origin (i.e., higher β-C in tropical regions). However, carotenoid composition was not explained by the climate of origin of the species according to Köppen–Geiger Climate Classification (Supplementary Table S3). Instead, floristic biome significantly explained metabolite composition of Fabaceae seeds (Supplementary Table S3 and **Figure 6B**). In particular, tropical groups differed significantly from temperate ones. In addition, β-C and other carotenoids from the β-branch in the pathway for carotenoids biosynthesis such as A, Z, and N (Cazzonelli, 2011) were among the metabolites that contributed most to the dissimilarities between floristic biomes (Supplementary Table S3). The significantly lower contents of those carotenoids (**Figure 6B**) in tropical vs. temperate biomes did not support the hypothesis that higher β-C contents play a role in attracting nitrogen-fixing symbionts in environments with unpredictable water availability. In contrast, functional group explained metabolite distribution among Fabaceae seeds, particularly for carotenoids (such as N, β-C, and L), which were present at higher concentrations in shrubs compared to other groups (Supplementary Figure S4B). As these are colored molecules, and β-C is additionally a precursor of odorous volatile compounds, their higher accumulation in shrub seeds could be related to a distinct dispersal strategy (i.e., attraction of certain vector animals) in shrubs compared to herbs and trees, and further research should be directed to test this hypothesis.

Although not totally independent from geographical distribution (i.e., most Papilionoideae are temperate, while Caesalpinioideae are preferentially tropical species), the distribution of carotenoids and tocochromanols showed clearly a phylogenetic basis (**Figures 1, 2, 3, 6** and Supplementary Table S3). The phylogenetic-based divergence in carotenoid and tocochromanol composition was particularly evident between Papilionoideae and Caesalpinioideae (**Figure 6A** and Supplementary Table S3), while Mimosoideae [former classification by Lewis (2005)] showed intermediate characteristics (Supplementary Figure S4A). The most remarkable differences included higher total carotenoid contents in Papilionoideae, in particular A, L, Z, and β-Car; absence of tocotrienols; γ- tocopherol as the main isoform of tocopherol; and higher Chl contents retained during maturation (**Table 1** and **Figure 6**). The divergence between Papilionoideae and Caesalpinioideae in carotenoid contents was also seen during seedling development (i.e., more pronounced accumulation of carotenoids in Caesalpinioideae vs. Papilionoideae species from *Stage* 3 to *Stage* 5, **Figure 8A**). The distinct lipophilic antioxidant composition of Papilionoideae seeds reported in this work is in agreement with recent data on the hydrophilic antioxidant homoglutahione which was shown to be restricted to the subfamily Papilionoideae within the Fabaceae (Colville et al., 2015) and with the higher phylogenetic distance of this clade from the rest (LPWG, 2017). The higher content of carotenoids, L, Z, and β-Car, with nutritional importance in human and livestock diets in seeds of Papilionoideae species could be of potential interest for food and agricultural industries.

## Conclusion

Fabaceae seeds contain all major carotenoids that are typical of photosynthetic tissues. Interestingly most of the species analyzed contain Lx, and its ratio to Chl trend to decrease during germination. Carotenoids’ correlation with chlorophyll contents and their proportional changes upon germination suggest they may act as structural compounds that support the assembly of the photosynthetic apparatus during seedling development. The lack of correlation between carotenoid and tocopherol contents could indicate that these two groups of lipophilic antioxidants play different roles in dry seeds. Interestingly, Papilionoideae seeds, to which all pulses of commercial importance belong, showed the highest contents of nutritionally valuable carotenoids, L, Z, and β-Car. The subfamily Papilionoideae showed very different patterns of carotenoid and tocochromanol composition compared to the subfamily Caesalpinioideae, having Papilionoideae higher L, β-C and Z contents, higher γ-T, lower β- and δ-T and no tocotrienols. Our results indicate that Papilionoideae is the most distinct subfamily and are thus in agreement with the current classification of Fabaceae (LPWG, 2017) and with recent data about the composition of the hydrophilic antioxidants, GSH and hGSH, in legume seeds (Colville et al., 2015). Our data further reveal that ecological traits, such as a high proportion of herbaceous and shrub species, and a major representation of temperate species within Papilionoideae, which are characters typical of this clade (Lewis, 2005), may also be related to the particular composition of lipophilic antioxidants in their seeds.

## Author Contributions

BF-M, JG-P, IK, and JB designed the study. BF-M and FM carried out the germination experiment and the HPLC analyses. AA and LC prepared the seed material. JG-P, LC, and BF-M drafted the manuscript. AA, LC, FM, and LM-F compiled information about ecology and phylogeny of the species. LM-F did the statistical analyses. All authors contributed to the discussion of the results and the writing of the work and approved the final version of the manuscript.

## Conflict of Interest Statement

The authors declare that the research was conducted in the absence of any commercial or financial relationships that could be construed as a potential conflict of interest.

## Footnotes

1 http://datadryad.org/resource/doi:10.5061/dryad.61pd6

2 www.ildis.org

3 tropical.theferns.info

4 www.worldagroforestry.org

5 www.floraiberica.es

## Abbreviations

α-C: α-carotene
A: antheraxanthin
β-C: β-carotene
CCDs: carotenoid cleavage dioxigenases
Chl: chlorophyll
GSH: glutathione
JBO: Jardín Botánico de Olarizu
L: lutein
Lx: lutein-epoxide
MSB: Millennium Seed Bank
N: neoxanthin
T: tocopherol
T3: tocotrienol
TotCarot: total carotenoids
TotTocochrom: total tocochromanols
V: violaxanthin
Z: zeaxanthin
