# Supplementary material for: Seed Carotenoid and Tocochromanol Composition of Wild Fabaceae Species Is Shaped by Phylogeny and Ecological Factors
Source: Front Plant Sci. 2017 Aug 24;8:1428. doi: 10.3389/fpls.2017.01428 (PMC5573840; doi:10.3389/fpls.2017.01428)

Supplementary Material

Table S1. Detailed information on the seeds of 50 Fabaceae species anaylsed. Subfamilies according to former (Lewis 2005) and current (LPWG 2017) classifications, numeric code for species in PCOA (Fig. 7), species name, number of replicates (n), seed bank and countries of origin of seed material are shown. The three species used for the germination experiments are indicated in bold font. Ecological factors used for the PCOA include: functional group, life form (according to Raunkiaers' classification), leaf phenology, climate native to the species (this was obtained according to species native geographical distribution. Then, the corresponding climate, following World Map of Köppen-Geiger Cliamte Classification updated (Kottek et al., 2006) and floristic biome were assigned (four floristic biomes were assigned to summarise original habitats of species).

Abbreviations in Table S1: MSB: Millennium Seed Bank (United Kingdom), JBO Jardín Botánico de Olarizu (Spain). A corresponds to equatorial climate, B to tropical, C temperate, D boreo-alpine. TemWet, temperate oceanic; TemDry, temperate mediterranean; TropDry, tropical with seasonal rain; TropWet: tropical humid.

Subfamily (Lewis 2005)	Subfamily (LPWG 2017)	Sp code	Species	*n*	SeedBank	Country	Seed Bank	Functional Group	Life Form	Leaf phenology	Climate	Florist biome
Caesalpinioideae	Cercidoideae (OtherFab)	1	*Adenolobus pechuelii* (Kuntze) Torre & Hillc.	*2*	MSB (RBG Kew)	Namibia	MSB (RBG Kew)	Shrub	Chamaephyte	Evergreen	B	TropD
Caesalpinioideae	Cercidoideae (OtherFab)	2	*Bauhinia reticulata* DC.	*3*	MSB (RBG Kew)	Burkina	MSB (RBG Kew)	Tree	Phanerophyte	Evergreen	AB	TropD
Caesalpinioideae	Detarioideae (OtherFab)	3	*Daniellia oliveri* (Rolfe) Hutch. & Dalziel	*5*	MSB (RBG Kew)	Burkina Faso	MSB (RBG Kew)	Tree	Phanerophyte	Deciduous	AB	TropD
Caesalpinioideae	Detarioideae (OtherFab)	4	*Colophospermum mopane* (Benth.) J.Léonard	*5*	MSB (RBG Kew)	Zimbabwe	MSB (RBG Kew)	Tree	Phanerophyte	Deciduous	B	TropD
Caesalpinioideae	Detarioideae (OtherFab)	5	*Afzelia africana* Pers.	*5*	MSB (RBG Kew)	Burkina Faso	MSB (RBG Kew)	Tree	Phanerophyte	Deciduous	A	TropD
Caesalpinioideae	Dialioideae (OtherFab)	6	*Dialium guineense* Willd.	*5*	MSB (RBG Kew)	Mali	MSB (RBG Kew)	Tree	Phanerophyte	Deciduous	AB	TropW
Caesalpinioideae	Caesalpinioideae	7	*Ceratonia siliqua* L.	*7*	MSB (RBG Kew)	Jordan	MSB (RBG Kew)	Tree	Phanerophyte	Evergreen	C	TemDr
Caesalpinioideae	Caesalpinioideae	8	*Pterogyne nitens* Tul.	*5*	MSB (RBG Kew)	Brazil	MSB (RBG Kew)	Tree	Phanerophyte	Deciduous	AC	TropW
Caesalpinioideae	Caesalpinioideae	9	*Caesalpinia mollis* (Kunth) Spreng.	*5*	MSB (RBG Kew)	Venezuela	MSB (RBG Kew)	Tree	Phanerophyte	Deciduous	A	TropD
Caesalpinioideae	Caesalpinioideae	10	*Cassia sieberiana* DC.	*5*	MSB (RBG Kew)	Burkina Faso	MSB (RBG Kew)	Tree	Phanerophyte	Deciduous	AB	TropD
Caesalpinioideae	Caesalpinioideae	11	*Peltophorum dubium* (Spreng.) Taub.	*4*	MSB (RBG Kew)	Brazil	MSB (RBG Kew)	Tree	Phanerophyte	Deciduous	C	TemW
**Caesalpinioideae**	**Caesalpinioideae**	**12**	***Erythrophleum africanum* (Benth.) Harms**	***5***	**MSB (RBG Kew)**	**Burkina Faso**	MSB (RBG Kew)	Tree	Phanerophyte	Deciduous	A	TropD
Mimosoideae	Caesalpinioideae	13	*Amblygonocarpus andongensis* (Oliv.) Exell & Torre	*5*	MSB (RBG Kew)	Botswana	MSB (RBG Kew)	Tree	Phanerophyte	Deciduous	A	TropD
Mimosoideae	Caesalpinioideae	14	*Entada abyssinica* A.Rich	*5*	MSB (RBG Kew)	Burkina Faso	MSB (RBG Kew)	Tree	Phanerophyte	Deciduous	ABC	TropD
Mimosoideae	Caesalpinioideae	15	*Neptunia oleracea* Lour.	*5*	MSB (RBG Kew)	Burkina Faso	MSB (RBG Kew)	Herb	Cryptophyte	Evergreen	B	TropW
Mimosoideae	Caesalpinioideae	16	*Dichrostachys cinerea* (L.) Wight & Arn. *subsp. africana* Brenan & Brummitt	*5*	MSB (RBG Kew)	Botswana	MSB (RBG Kew)	Tree	Phanerophyte	Deciduous	B	TropD
**Mimosoideae**	**Caesalpinioideae**	**17**	***Leucaena leucocephala* (Lam.) de Wit**	***5***	**MSB (RBG Kew)**	**Yemen**	MSB (RBG Kew)	Tree	Phanerophyte	Evergreen	A	TropD
Mimosoideae	Caesalpinioideae	18	*Parkia biglobosa* (Jacq.) G.Don	*5*	MSB (RBG Kew)	Burkina Faso	MSB (RBG Kew)	Tree	Phanerophyte	Deciduous	ABC	TropD
Mimosoideae	Caesalpinioideae	19	*Paraserianthes lophantha* (Willd.) I.C.Nielsen	*6*	MSB (RBG Kew)	South Africa	MSB (RBG Kew)	Tree	Phanerophyte	Evergreen	C	TemDr
Mimosoideae	Caesalpinioideae	20	*Faidherbia albida* (Delile) A.Chev.	*5*	MSB (RBG Kew)	Sudan	MSB (RBG Kew)	Tree	Phanerophyte	Deciduous	B	TropW
Mimosoideae	Caesalpinioideae	21	*Acacia angustissima* (Mill.) Kuntze	*5*	MSB (RBG Kew)	USA	MSB (RBG Kew)	Tree	Phanerophyte	Evergreen	ABC	TropD
Mimosoideae	Caesalpinioideae	22	*Havardia albicans* (Kunth) Britton & Rose	*6*	MSB (RBG Kew)	Mexico	MSB (RBG Kew)	Tree	Phanerophyte	Evergreen	AB	TropD
Mimosoideae	Caesalpinioideae	23	*Havardia pallens* (Benth.) Britton & Rose	*3*	MSB (RBG Kew)	Mexico	MSB (RBG Kew)	Tree	Phanerophyte	Evergreen	AB	TropD
Papilionoideae	Papilionoideae	24	*Xanthocercis zambesiaca* (Baker) Dumaz-le-Grand	*5*	MSB (RBG Kew)	Zimbabwe	MSB (RBG Kew)	Tree	Phanerophyte	Evergreen	ABC	TropD
Papilionoideae	Papilionoideae	25	*Sophora arizonica* S. Watson	*5*	MSB (RBG Kew)	USA	MSB (RBG Kew)	Tree	Phanerophyte	Deciduous	B	TropD
Papilionoideae	Papilionoideae	26	*Luetzelburgia auriculata* (Allemão) Ducke	*5*	MSB (RBG Kew)	Brazil	MSB (RBG Kew)	Tree	Phanerophyte	Evergreen	A	TropD
Papilionoideae	Papilionoideae	27	*Pterocarpus erinaceus* Poir.	*6*	MSB (RBG Kew)	Burkina Faso	MSB (RBG Kew)	Tree	Phanerophyte	Deciduous	ABC	TropD
**Papilionoideae**	**Papilionoideae**	**28**	***Podalyria myrtillifolia* (Retz.) Willd.**	***5***	**MSB (RBG Kew)**	**South Africa**	MSB (RBG Kew)	Tree	Phanerophyte	Evergreen	C	TemDr
Papilionoideae	Papilionoideae	29	*Virgilia oroboides* (P.J.Bergius) T.M.Salter	*5*	MSB (RBG Kew)	South Africa	MSB (RBG Kew)	Tree	Phanerophyte	Semideciduous	C	TemDr
Papilionoideae	Papilionoideae	30	*Lupinus angustifolius* L.	*4*	MSB (RBG Kew)	Greece	MSB (RBG Kew)	Herb	Therophyte	Deciduous	C	TemDr
Papilionoideae	Papilionoideae	31	*Genista hispanica* L.	*5*	JBO (Vitoria)	Spain	BGJBO (Vitoria)	Shrub	Chamaephyte	Evergreen	C	TemDr
Papilionoideae	Papilionoideae	32	*Genista scorpius* (L.) DC.	*7*	JBO (Vitoria)	Spain	BGJBO (Vitoria)	Shrub	Phanerophyte	Evergreen	C	TemDr
Papilionoideae	Papilionoideae	33	*Genista tinctoria* L.	*5*	JBO (Vitoria)	Spain	BGJBO (Vitoria)	Shrub	Chamaephyte	Evergreen	C	TemW
Papilionoideae	Papilionoideae	34	*Centrosema pubescens* Benth.	*5*	MSB (RBG Kew)	Sierra Leone	MSB (RBG Kew)	Herb	Hemicryptophyte	Evergreen	A	TropD
Papilionoideae	Papilionoideae	35	*Philenoptera violacea* (Klotzsch) Schrire	*2*	MSB (RBG Kew)	Zimbabwe	MSB (RBG Kew)	Tree	Phanerophyte	Semideciduous	ABC	TropW
Papilionoideae	Papilionoideae	36	*Galactia striata* (Jacq.) Urb.	*3*	MSB (RBG Kew)	Brazil	MSB (RBG Kew)	Herb	Chamaephyte	Evergreen	A	TropD
Papilionoideae	Papilionoideae	37	*Bituminaria bituminosa* (L.) C.H.Stirt.	*5*	JBO (Vitoria)	Spain	BGJBO (Vitoria)	Shrub	Hemicryptophyte	Evergreen	C	TemDr
Papilionoideae	Papilionoideae	38	*Vigna adenantha* (G.Mey.) Maréchal, Mascherpa & Stainier	*5*	MSB (RBG Kew)	Central African Republic	MSB (RBG Kew)	Herb	Chamaephyte	Evergreen	B	TropW
Papilionoideae	Papilionoideae	39	*Sesbania sesban* (L.) Merr. *subsp. sesban*	*4*	MSB (RBG Kew)	Mali	MSB (RBG Kew)	Tree	Phanerophyte	Evergreen	AB	TropD
Papilionoideae	Papilionoideae	40	*Anthyllis vulneraria* L.	*2*	JBO (Vitoria)	Spain	BGJBO (Vitoria)	Herb	Hemicryptophyte	Deciduous	C	TemDr
Papilionoideae	Papilionoideae	41	*Dorycnium pentaphyllum* Scop.	*5*	JBO (Vitoria)	Spain	BGJBO (Vitoria)	Shrub	Chamaephyte	Evergreen	C	TemDr
Papilionoideae	Papilionoideae	42	*Lotus corniculatus* L.	*5*	JBO (Vitoria)	Spain	BGJBO (Vitoria)	Shrub	Hemicryptophyte	Evergreen	C	TemDr
Papilionoideae	Papilionoideae	43	*Lotus pedunculatus* Cav.	*5*	JBO (Vitoria)	Spain	BGJBO (Vitoria)	Herb	Hemicryptophyte	Evergreen	C	TemW
Papilionoideae	Papilionoideae	44	*Glycyrrhiza echinata* L.	*6*	MSB (RBG Kew)	Lebanon	MSB (RBG Kew)	Shrub	Geophyte	Evergreen	D	TemDr
Papilionoideae	Papilionoideae	45	*Astragalus glycyphyllos* L.	*5*	JBO (Vitoria)	Spain	BGJBO (Vitoria)	Herb	Geophyte	Evergreen	C	TemW
Papilionoideae	Papilionoideae	46	*Onobrychis conferta subsp. argentea* (Boiss.) Guitt. & Kerguelen	*5*	JBO (Vitoria)	Spain	BGJBO (Vitoria)	Herb	Hemicryptophyte	Evergreen	C	TemDr
Papilionoideae	Papilionoideae	47	*Ononis spinosa* L.	*5*	JBO (Vitoria)	Spain	BGJBO (Vitoria)	Herb	Chamaephyte	Evergreen	C	TemDr
Papilionoideae	Papilionoideae	48	*Melilotus albus* Medik.	*3*	JBO (Vitoria)	Spain	BGJBO (Vitoria)	Herb	Hemicryptophyte	Deciduous	C	TemW
Papilionoideae	Papilionoideae	49	*Trifolium pratense* L.	*5*	JBO (Vitoria)	Spain	BGJBO (Vitoria)	Herb	Hemicryptophyte	Evergreen	C	TemW
Papilionoideae	Papilionoideae	50	*Lathyrus pratensis* L.	*5*	JBO (Vitoria)	Spain	BGJBO (Vitoria)	Herb	Hemicryptophyte	Evergreen	C	TemW

Table S2. Pearson's correlation coefficients for each variable used in the Principal Coordinates Analysis (PCOA). Significant correlations with each axis are highlighted in bold.

	**N**	**V**	**Lx**	**A**	**L**	**Z**	**β-C**	**α-T**	**γ-T**	**TotChl**	**β+δ-T**	**TotT3**
**PCO1**	**0.91**	**0.86**	0.71	**0.85**	**0.84**	0.79	**0.86**	0.47	0.00	**0.81**	-0.44	-0.18
**PCO2**	-0.08	0.06	0.12	-0.13	0.01	-0.14	-0.16	**0.64**	**-0.78**	0.01	-0.22	**0.58**

Table S3 Statistical significance of multivariate analyses. R and *p* values (in brackets) are shown for Global (all categories within each Factor) and Pairwise (couples of categories within each Factor) Analysis Of SIMilarity (ANOSIM). The contribution of individual variables to significant dissimilarity among categories were provided by SIMilarity PERcentages breakdown procedure (SIMPER) and are shown as percentages. Significant R and *p* values are highlighted in bold.

FACTORS	Global Test R (p)	Pairwise Tests R (p)	SIMPER- Between groups dissimilarity contribution %
**PREVIOUS SUBFAMILY CLASSIFICATION**	0.09 (0.086)		
**NEW SUBFAMILY CLASSIFICATION**	***0.104 (0.045)***		
Other, Caesalpinioideae		0.01 (0.434)	
Other, Papilionoideae		0.177 (0.090)	
Caesalpinioideae, Papilionoideae		**0.104 (0.033)**	TotT3 (10.96%); A (8.83%); TotChl (8.81%); Z (8.81%); L (8.30%)= 45.72%
**FUNCTIONAL GROUP**	***0.200 (0.006)***		
Shrub, Tree		**0.348 (0.005)**	β-C (12.52%); N (12.32%); A (10.80%); Z (9.75%); V (9.49%)=54.89%
Shrub, Herb		0.034 (0.279)	
Tree, Herb		**0.144 (0.041)**	Z (10.67%); α-T (10.17%); A (9.97%); γ-T (9.60%); Lx (9.49%)= 49.9%
**LIFE FORM**	***0.223 (0.012)***		
Chamaephyte, Phanerophyte		**0.463 (0.001)**	β-C (10.69%); A (10.38%); N (9.88%); β+δ T (9.79%); Lx (9.43%)= 50.17%
Chamaephyte, Cryptophyte		-0.374 (0.875)	
Chamaephyte, Therophyte		-0.32 (0.875)	
Chamaephyte, Hemicryptophyte		0.076 (0.167)	
Chamaephyte, Geophyte		-0.156 (0.750)	
Phanerophyte, Cryptophyte		-0.183 (0.751)	
Phanerophyte, Therophyte		0.024 (0.323)	
Phanerophyte, Hemicryptophyte		**0.245 (0.017)**	Z (11.72%); A (10.96%); N (9.76%); γ-T (8.75%); TotChl (8.73%)= 49.92%
Phanerophyte, Geophyte		-0.164 (0.702)	
Cryptophyte, Hemicryptophyte		-0.414 (0.900)	
Cryptophyte, Geophyte		-1.00 (1.000)	
Therophyte, Hemicryptophyte		-0.340 (0.900)	
Therophyte, Geophyte		0.000 (0.667)	
Hemicryptophyte, Geophyte		-0.126 (0.545)	
**LEAF TYPE**	0,053 (0.149)		
**CLIMATE**	0.045 (0.258)		
**FLORISTIC BIOME**	***0.174 (0.007)***		
TropDry, TropWet		-0.066 (0.670)	
TropDry, TemDry		**0.226 (0.002)**	A (11.69%); Z (10.95%); N (9.40%); V (9.24%); β-C (8.98%)= 50.26%
TropDry, TemWet		**0.345 (0.009)**	TotT3 (13.52%); γ-T (9.77%); TotChl (8.94%); N (8.73%); β-C (8.16%)=49.12%
TropWet, TemDry		0.140 (0.128)	
TropWet, TemWet		0.119 (0.118)	
TemDry, TemWet		0.056 (0.252)	
**AQUATIC PREFERENCE**	0.037 (0.355)		

Fig. S1 Occurrence (i.e. % of the species where each metabolite was found) (A), and average content of carotenoids (B) in Fabaceae seeds. Bars represent mean ± SE (n=50 species)

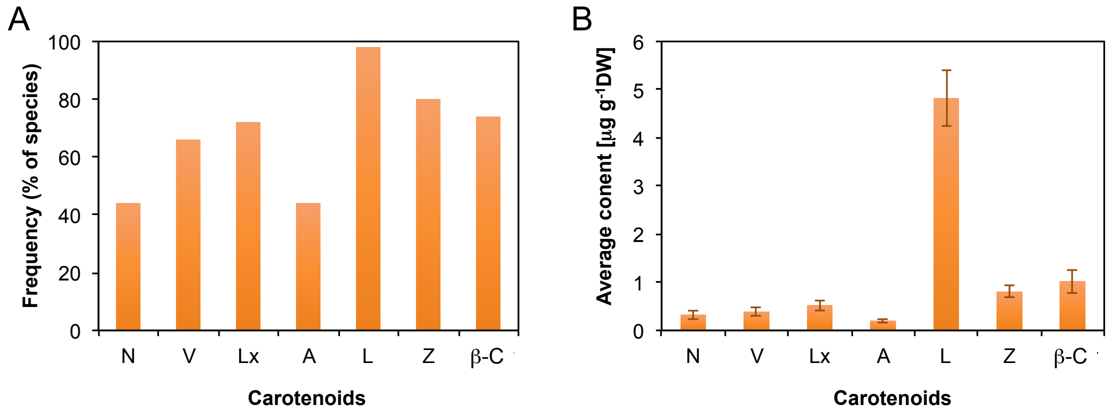

Fig. S2 Occurrence (i.e. % of the species in which each metabolite was found) (A), and average content of tocochromanols (B) in Fabaceae seeds. Bars represent mean ± SE (n=50 species)

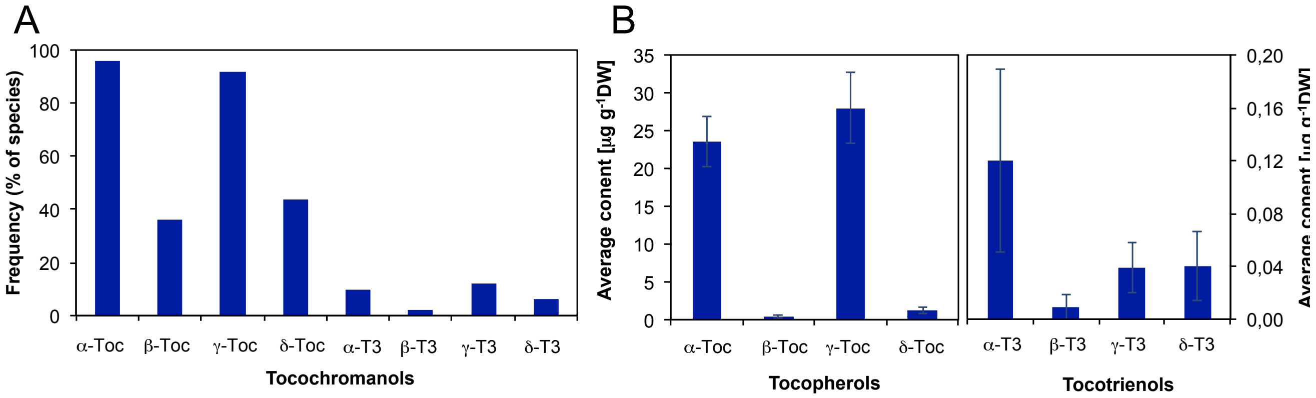

Fig. S3 Relationship between total carotenoids and total tocopherols contents of Fabaceae seeds. Each data point represents the average of a single species. Carotenoids and tocopherols contents were positively correlated within the Caesalpinioideae subfamily. Fit to a linear regression model and R^2 is shown for this case ($P<0.05$).

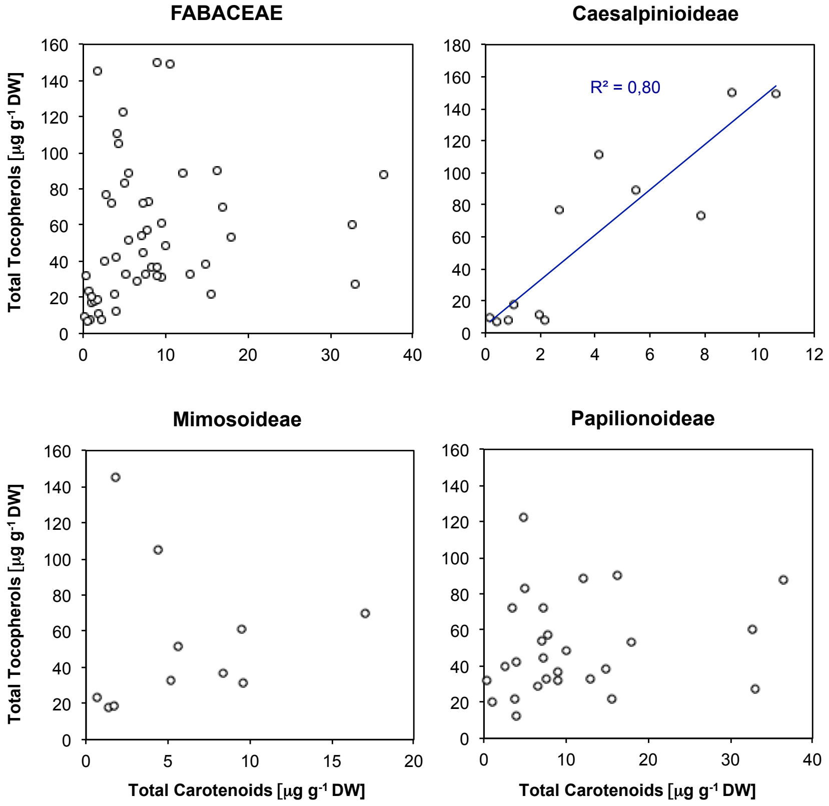

Fig. S4 Box plot illustrating the contents of individual metabolites for (A) different subfamilies (according to former classification, Lewis 2005) and (B) different functional groups. Boxes cover 50% of the data. Central lines represent the medians and whiskers represent the minimum and maximum values among non-atypical data. Significant differences between categories are indicated with letters ($P<0.05$).

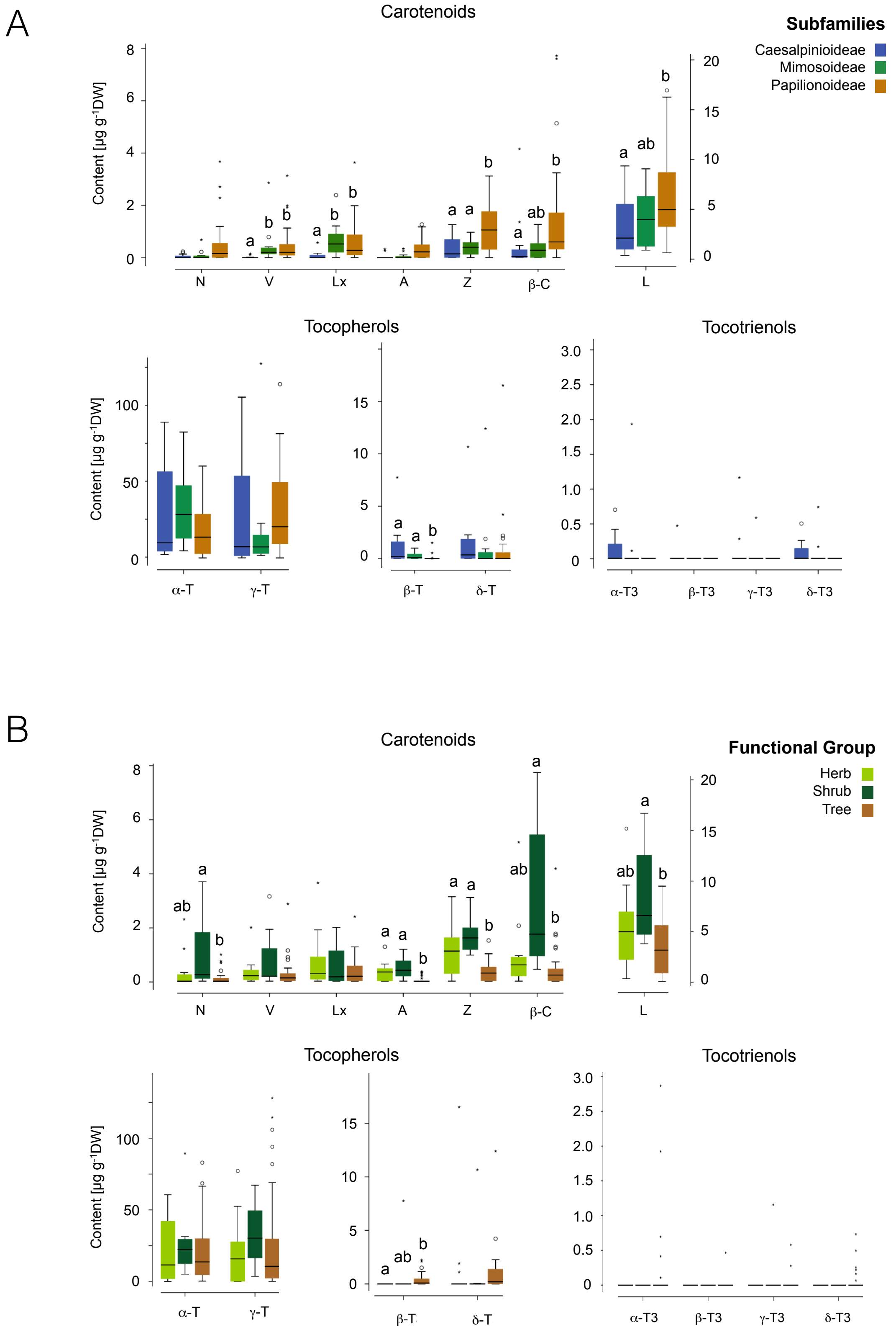

Fig. S5 A: Change in Chl total content during germination and seedling establishment. B: Change in the absolute content of carotenoids at Time 5 of germination (hypocotyl longer than seed) compared to Time 1 (dry seed).

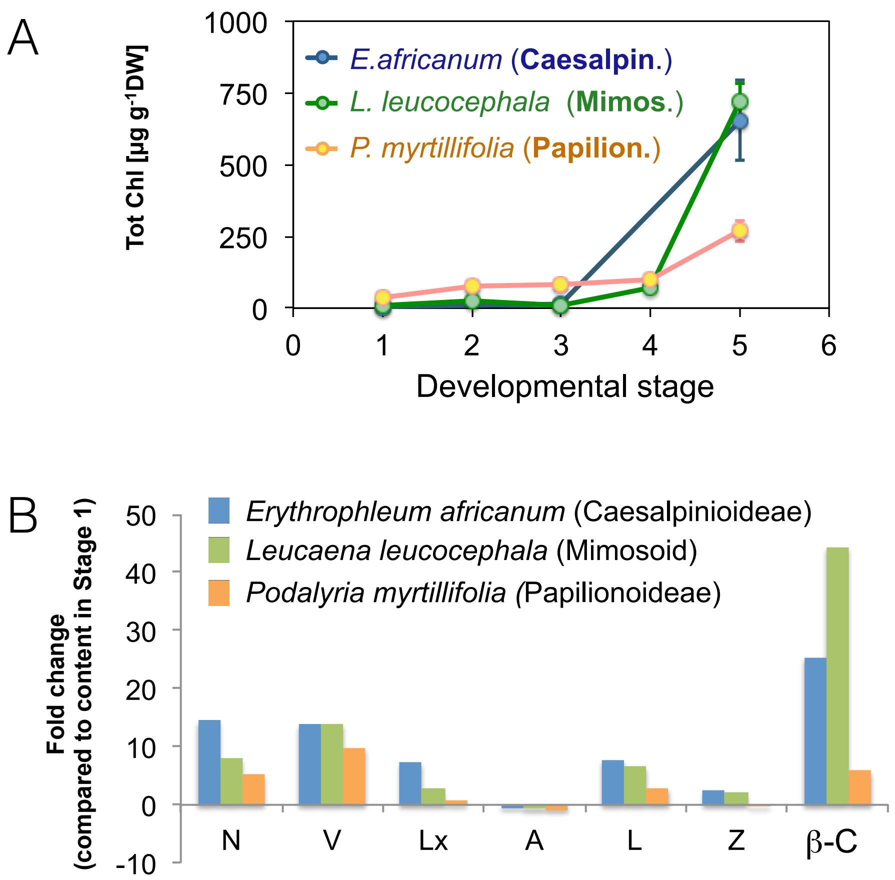

Supplement: Supplementary file 1 [file Data_Sheet_1.pdf]
